# Application of hyperspectral remote sensing for supplementary investigation of polymetallic deposits in Huaniushan ore region, northwestern China

**DOI:** 10.1038/s41598-020-79864-0

**Published:** 2021-01-11

**Authors:** Yu-qing Wan, Yu-hai Fan, Mou-shun Jin

**Affiliations:** 1 Geological Exploration Institute of Aerial Photogrammetry and Remote Sensing Bureau, Xi’an, 710199 People’s Republic of China; 2 School of Earth Science and Land and Resources, Chang’an University, Xi’an, 710045 People’s Republic of China; 3 Xi’an Center of Geological Survey, Xi’an, 710054 People’s Republic of China

**Keywords:** Solid Earth sciences, Engineering, Optics and photonics

## Abstract

A gold–silver–lead–zinc polymetallic ore was selected in Huaniushan, Gansu Province as the study area. Hyperspectral aerial images as the primary information source, ground spectrum tests, and sampling analysis were used as auxiliary techniques. They were combined with large-scale mineral and geological maps and other high-resolution satellite remote sensing images. Hyperspectral remote sensing classification identification and quantitative analysis methods were used to study the main mineral resources and rock mass occurrence. Finally, deposit distribution information was extracted and validated. The results showed that the effective classification methods by hyperspectral images were spectral angle mapping, minimum noise fraction transform, and mixed tuned matched filtering. Based on the ground survey, combined with sampling analysis, the accuracy of classification was 80%. The recognition rate of the main ore body—the iron-manganese cap lead–zinc oxide ore—was as high as 81%. This research showed that hyperspectral remote sensing in this mining area has excellent demonstration effects and is worth completing and supplementing original mineral and geological maps. The targets are important areas for detailed follow-up on mineral resource exploration.

## Introduction

Minerals and rocks generally have diagnostic spectral absorption bands in the spectral range of 400–2500 nm. Hyperspectral remote sensing can effectively capture spectral characteristics. Mineral composition information can be inverted and identified according to the spectrum^1–5^. As a result, rock and mineral classification and mapping, and resource exploration can be performed^6–9^. Over the years, experts and scholars around the world have conducted a series of application tests, with great expectations for the application and effectiveness of hyperspectral remote sensing in mineral exploration^10–12^. Some spectral libraries and expert system software have been established to map minerals using hyperspectral images^13–15^. For example, each pixel spectrum in the AVIRIS image was compared to the spectra of more than 300 substances (including minerals, mineral mixtures, vegetation, water, snow, and artificial materials) in the USGS spectral library for discrimination and classification^2^. A compiled lithological map was drawn in a subarctic region (Cape Smith Belt, Nunavik, Canada) by geologists using an iterative spectral unmixing approach with image spectral end members extracted from SWIR and other images. This was based on locations defined by previous works in the study area and field mapping information. Depending on the spectral characteristics of different rocks, researchers can extract the information about various rock types and apply hyperspectral remote sensing from qualitative analysis to quantitative recognition^16^ in southern Namibia. They mapped rocks and altered minerals in the Wambed mining area. They successfully identified sericite in the Weipin Dyke and hornblende in the iron-magnesian ultramafic rocks such as gabbro and gabbro-norite. In Zhangjiakou, Hebei Province, Chinese experts^17^ extracted rock information by image processing according to spectral characteristics of different rocks and applied hyperspectral remote sensing to geoscience from qualitative analysis to quantitative identification. In the Liuyuan Fangshankou area of Beishan, Gansu Province, geologists conducted solid mineral exploration^18^ using aerial hyperspectral remote sensing technology combined with conventional geological investigations. Seven prospecting targets were found, and noticeable prospecting results were obtained.

Many practices^19–23^ have demonstrated that hyperspectral data play an increasingly important role in rock and mineral identification, solid minerals, and oil and gas exploration^24–26^. Hyperspectral remote sensing technology has obvious advantages in prospecting solid mineral^27,28^. First, through mineral mapping, altered minerals can be quickly extracted over a wide area. Second, through the integration of images and spectra, altered minerals and their types can be identified by spectrum more precisely. The location, scale, shape, control factors, and distribution characteristics of an image can be visualized^18^.

However, spectral curves change with type, the mineral-to-rock combination ratio, and the observation scale (spatial resolution)^1,5^. Analysis of several commonly used spectral libraries, such as USGS-min, JPL, JHU, IGCP-264, and ASTER, reveals that some minerals spectra vary considerably from one spectral library to another^29^. Since there are different types of curves in the same library, we explained some factors that affect the spectral properties of minerals and rocks and pointed out the precautions for establishing a spectral library.

Researchers have adopted various spectral information extraction methods in hyperspectral remote sensing applications and found that different methods have their advantages and disadvantages^30,31^. Different application targets require different mineral identification and mapping schemes. Various information extraction technologies, including multi-source remote sensing information fusion^32–36^, are also required. Finally, it was pointed out that the development and application of the hybrid method and the fusion application from the visible band to the microwave band are more important in the future. Many researchers^8,9,30^ systematically summarized the geological application modes and mineral mapping technology flow of hyperspectral mineral identification, suggested some application examples, and proposed research directions for hyperspectral mineral mapping. Researchers^26^ integrated spectral enhancement techniques such as Principal Component Analysis (PCA), Independent Component Analysis transformation, and different MLAs to accurately map rock types. They achieved an Overall Accuracy (OA) of 85.48% and a Kappa Coefficient (k) of 0.83.

At present, aerial hyperspectral remote sensing images are relatively expensive, the imaging period is generally long (from 9:30 a.m. to 3:30 p.m. local time), and data preprocessing is also time-consuming. Therefore, it cannot be widely used and affects the promotion of geological exploration technology. Because of the problems in aerial hyperspectral remote sensing^37,38^, some scholars have systematically summarized the geological application modes of hyperspectral mineral identification and the flow of mineral mapping techniques. They have also provided demonstrations for several applications and proposed directions for recent research in hyperspectral mineral mapping. Geological exploration applications are becoming more and more popular due to breakthroughs in satellite hyperspectral remote sensing data.

The research area in this paper is an important polymetallic metallogenic belt in northwestern Gansu Province, China. The mining history is more than 50 years in this region, and mineral resources tend to be exhausted. To find the follow-up resources, geologists have conducted supplementary exploration works and performed aerial hyperspectral remote sensing prospecting studies. The goals of this research was (1) to study the applicability of hyperspectral remote sensing prospecting and find new target regions for deposits, (2) to propose rational workflow and technical methods for using hyperspectral remote sensing and high-resolution multispectral remote sensing for ore prospecting, and (3) to check and edit maps at a scale of 1:10,000 from conventional geological surveys.

### Metallogenic background in Huaniushan polymetallic mine

Huaniushan gold-silver-lead–zinc metallogenic area is located southwest of Beishan at the junction of Gansu and Xinjiang. Since the Middle Proterozoic, the Huaniushan mining area has undergone many stages of tension, rifting, Hercynian compressional orogeny, continental crust denudation, and surface planation^38^, and intense tectonic and magmatic activities from the Indosinian period. These processes have resulted in very complex structural patterns of current fold and fault structures. The exposed strata in the mining area are mainly the upper series of the Jixian System^39^, followed by intermediate sequences of the Ordovician system. The upper series of the Jixian System can be further divided into three lithological sections^40^. The Jxp^3a^ lithological section is exposed to the south of the Huaniushan Huaxitan Fault, the main lithology of which is slate, phyllite, and metasandstone intercalated with marble. The Jxp^3b^ lithological section is widely distributed, a slightly metamorphosed set of argillaceous silty sand and fine clastic rock formation. These strata are the main occurrence of hydrothermal copper, molybdenum, tungsten, gold, and silver mineralization associated with granite. The Jxp^3c^ lithological section is the main ore-bearing exhalative sedimentary deposit with gold, silver, lead, and zinc in the mining area. This section contains ore-bearing marble with phyllite in the first, second, and fourth mining areas and ore-bearing basic volcanic rocks with carbon-rich siliceous slate, phyllite, and marble lens in the third mining area.

The lithology of the Middle Ordovician is mainly sandstone, slate, marble, and sandstone slate with crystalline limestone, with fault contact with the Jixian System. Magmatic activities in the mining area are intense and frequent, mainly manifested by the late Mesoproterozoic submarine volcanic eruptions and magmatic intrusions in the early and middle of the Variscan orogeny and Indosinian.

Since its discovery in the late 1950s, this deposit has been identified as a medium-sized silver-lead–zinc deposit after detailed investigation and exploration. But after decades of mining, it was ranked as a resource-exhausted mine mine. A few years ago, researchers^18,38^ conducted hyperspectral remote sensing research in the Huaniushan polymetallic ore. They made ore exploration predictions around the Huaniushan lead–zinc deposit by studying the types of hyperspectral mineralization and alteration and the characteristics of their combinations^18,41^. The ore-forming geological conditions in this area are promising. The ore-forming type is typical, and the alteration type is diverse. Bedrock exposure is comprehensive, the remote sensing geological features are obvious^42,43^, and previous studies are credible. High-quality hyperspectral images of the mining area are suitable for research and further prospecting of hyperspectral remote sensing techniques for polymetallic ores.

### Hyperspectral remote sensing technology in Huaniushan polymetallic mining area

#### Data source

In this study, we collected geological and mineral data, including the existing geological maps of the Huaniushan gold and silver polymetallic ore (1:10,000 scale), reports on the metallogenic model, and exploratory predictions of gold and silver polymetallic ores in the region^38,39^. 8-band Worldview-2 images were also collected to meet the needs of interpretation and field spectrum testing. The spatial resolution of Worldview-2 image is 0.5 m, which can be used for large-scale comprehensive remote sensing surveys of mineral resources. The combination and enhancement of bands allow extracting structural, stratigraphic, and lithological information related to ore formation and control. Such features are convenient for comprehensive studies.

CASI/SASI airborne hyperspectral images are the primary data source for this study, including two adjacent scanlines and 137 bands. There are 36 bands within 380–1050 nm with a ground resolution of 1 m, and 101 bands within 950–2450 nm with a ground resolution of 2.2 m. Hyperspectral images have undergone rigorous spectral calibration, geometric correction, and mosaicking^40,42,43^.

### Lithology and mineral information enhancement processing and hyperspectral mapping

1. How to enhance lithological and mineral information in multispectral images.

Worldview-2 images were processed by band operation and PC transformation to identify and extract relevant mineralization information or geological bodies with mineralization. The ore-forming and controlling elements, altered minerals, and mineralized alteration zones of metal ores were enhanced.

(2) Mineral mapping and classification by hyperspectral remote sensing.

In general, there are two groups of effective methods. The first includes minimum noise separation (MNF) and PC transformation^26^ to reduce the dimensions of hyperspectral images and compress the information into an image with a specified dimension (n). We can use n-D and PPI tools to analyze the MNF result image^42^ and find pure pixels in the spectrum. These pure pixels become the sample end members, extract the end members spectral curves, and establish a spectral library for classification. The resulting MNF images have been shown to play an important role in rock recognition and boundary delineation, especially for the main local minerals. The second group includes mineral pixel analysis and classification based on spectral libraries such as spectral angle mapping (SAM^14^), spectral feature fitting, spectral separation (UNMIX^31,33^), and mixture tuned matched filtering (MTMF). Of these methods, SAM and MTMF were the most practical in the study.

#### Field investigation, verification, sampling analysis

To validate the information obtained from hyperspectral and multispectral images, we referred to 1:10,000 mineral and geological maps to analyze, pre-identify, and screen mineralized anomalous alteration regions before fieldwork. Then, we selected target areas combined with landform and traffic conditions, deployed field investigation verification spots and sampling points, and ensured that the distribution could cover all types of anomalies.

The main contents of the survey were the field investigation of lithological types and mineral compositions in ten anomalous mineralized alteration areas (zones), in situ rock mass measurements, and sample collection. At the same time, we observed and analyzed linear structures, circular structures, massive structures, ductile shear zones, and volcanic rock structures. The first objective of field investigation was to study the authenticity of remote sensing information. The second objective was to study the outcrops of typical ore bodies, analyze ore-forming conditions, ore-controlling factors, and mineralization laws, explore the genesis of ore deposits, and summarize regional metallogenic models.

Survey and verification means included field observations, GPS positioning, spectral testing, sampling, and more. We used GPS to locate observation points and rock mass and place observation lines. A combination of crossing and the tracing methods was used to track and observe the strike of common vertical rock strata or structural belts along the strike of structural lines and the direction of significant geomorphic changes. Significant geological bodies, mineralized belts, fault belts, and ore outcrops were traced along strikes to control boundaries. An ASD FieldSpec 4 spectrometer was used to measure the spectra of known ores and surrounding rocks based on the geological map of minerals. These are used as standard spectra for laboratory analysis.

We simultaneously collected surface outcrop samples during the field spectrum test, including in situ weathered and bedrock samples. There were 84 samples in total. Most samples were from the ore bodies in the mineralized alteration area, and some were known as typical granitic bodies. The purpose was to verify the accuracy of the 1:10,000 mineral geological maps and fully understand the spectral characteristics of typical rocks and ores.

#### Rock ore identification

After preliminary identification of samples and their field spectra, 45 uncertain ore (rock) samples were selected and sent to the Xi'an Center of Geological Survey to determine their names and mineral components using thin sections observation. Two types of tests were done in this process. The first was to identify the mineral composition under a microscope and accurately calculate the content (%) of each component. The second was to measure the spectra of fresh and weathered surfaces formed during the sample slicing process to correctly understand the phenomenon of different spectra with the same mineral composition and different mineral compositions with the same spectrum in the follow-up analysis. The purpose was to find sensitive bands in hyperspectral images, provide a basis for refining and combining classifications, and provide a theoretical basis for optimizing and improving accurate classification and recognizing hyperspectral images.

### Effective method and typical application effect of hyperspectral images in polymetallic geological exploration

The hyperspectral image used had a total of 137 bands. According to the statistical analysis of brightness, contrast, and signal-to-noise ratio of each band, 92 effective bands were selected for the subsequent analysis and processing after spectral calibration.

#### Spectral angle mapping (SAM)

In n-dimensional space (n is the band number of image), pixel classification is determined by the similarity between the pixel spectrum and the reference spectrum (standard spectrum). In this method, the n-band spectral reflectance is treated as a vector in n-dimensional space. By calculating the "spectral angle" between a given pixel and an end member, the matching degree of both is determined. The less the angle, the higher the similarity. The classification of the given pixel is then determined according to the similarity threshold given by the user. SAM is based solely on the "direction" of the spectral vector, without considering the "length" of the vector. Therefore, this classification method is not particularly sensitive to differences in pixel brightness. It is not easy to misclassify the same object with different slope directions and slopes because it is not sensitive to ground lighting.

**Figure 1:**
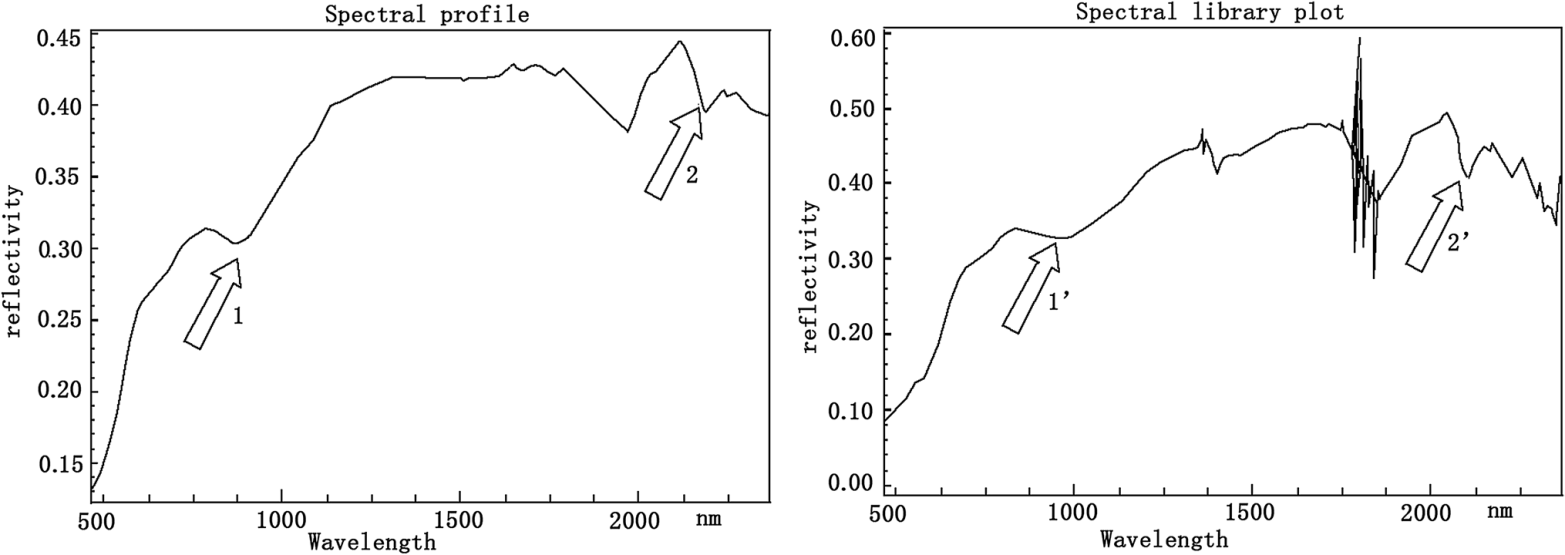
Suspected spectrum (left) of γ _5_^1a^ collected from the image and field measured spectrum (right).This figure is generated by Yu-Hai Fan, using CorelDRAW X6 created by the CorelDRAW Team under an open license (http://www.coreldraw.com/cn/product/graphic-design-software/).

In the actual operation process, the first step is to establish a suitable spectral library. There are two methods to build it. The first is based on the field spectrum of the study area. The objective is to analyze and verify that the spectral curve from the field object is comparable to that from the hyperspectral image. As shown in Fig. 1, the measured spectrum is on the right, and the spectrum of γ5^1a^ collected at the same position is on the left. It can be seen that the similarity between the two spectra is relatively high. Arrows at 890 nm, and 2200 nm correspond to trough positions. After excluding the strong absorption by water vapor around 1400 nm and 1850 nm, the two spectral curves are almost identical. Spectral preprocessing and resampling ensure uniform band count, spectral interval, and wavelength range in the hyperspectral image. The process of this method is relatively complex, and the effect is not as good as the next method.

The second method refers to large-scale geological mineral maps, selects typical strata and rock mass units that can be seen in hyperspectral images, draws training areas (ROIs), and statistically obtains the spectral curves of each ROI group (If there are several ROIs in the same type of rocks, they are classified into one ROI group). This method also establishes the spectrum library. There are 14 ROI groups in this area: First granite (Indosinian) subclass 1 and subclass 2, hornstone, plagiogranite granite porphyry granite, phyllite, marble, skarn, schist, iron-manganese cap lead–zinc oxide orebody, macular vein, second granodiorite, altered granite by thermal contact hornstone, and granite vein.

After establishing the spectral library, we used ENVI software to match each i-spectrum (i = 1 ~ 14) in the spectrum library with the corresponding spectrum of each pixel (P (x, y)) on the image and obtained 14 SAMs. The final classification result of P (x, y) belongs to the class with SAM's minimum value.

We used SAM to classify hyperspectral image and obtained a lithological classification map. The user accuracy analysis (Table 1) and the error matrix of SAM results are calculated using the 1:10,000 geological mineral map as the benchmark map. The meaning of user accuracy is that in the classification result, the probability that the sample matches the actual situation indicates the reliability of the SAM results (overall accuracy = 65.37%; Kappa coefficient = 0.59).

**Table 1:** accuracy statistics of SAM results (user accuracy).

Stratum name	Category (stratigraphic code)	User accuracy (%)
First granite (Indosinian) subclass 1	γ_5_^1a^	85.98
First granite (Indosinian) subclass 2	γ_5_^1b^	93.73
Horn stone	Hs	88.38
Plagioclase granite porphyry	γοπ	71.85
Granite	γπ	60.26
Phyllite	Ph	66.12
Marble	Mb	37.11
Skarn	Sk	16.73
Schist	Sch	60.71
Iron-manganese cap lead–zinc oxide orebody	G	81.03
Macular vein	χ	12.08
SECOND grano diorite	γδ_4_^2b^	39.67
Altered granite by thermal contact hornstone	Jxp_3_^c^	87.63
Granite vein	γ	1.56

As shown in Table 1, the recognition rate of γ, χ, γδ_4_^2b^, Sk, Mb strata, and rock masses is not high, and the recognition rate of granite vein γ and lamprophyre vein χ is particularly low. The vein γ is exaggerated on the map because it is too small and important in the geological mineral map. Most pixels in and around the vein are a mixture of hyperspectral images with unobtrusive spectral characteristics. It is encouraging that the SAM recognition rate of the main ore body of Pb–Zn oxidized ore with Fe–Mn cap (G-type) is as high as 81%. Therefore, G-type spots in the classification result must be the focal area for future exploration.

#### Minimum noise fraction transform and principal components analysis transform (PCA)

After MNF and PCA processing, 10 to 20 effective components can be generated. Many changes can be obtained by sequentially analyzing the image features and statistical data for each component and combining them with the false color composition. The existing 1:10,000 geological mineral map shows that hyperspectral remote sensing effectively identifies intrusive rock and invaded rock.

**Figure 2:**
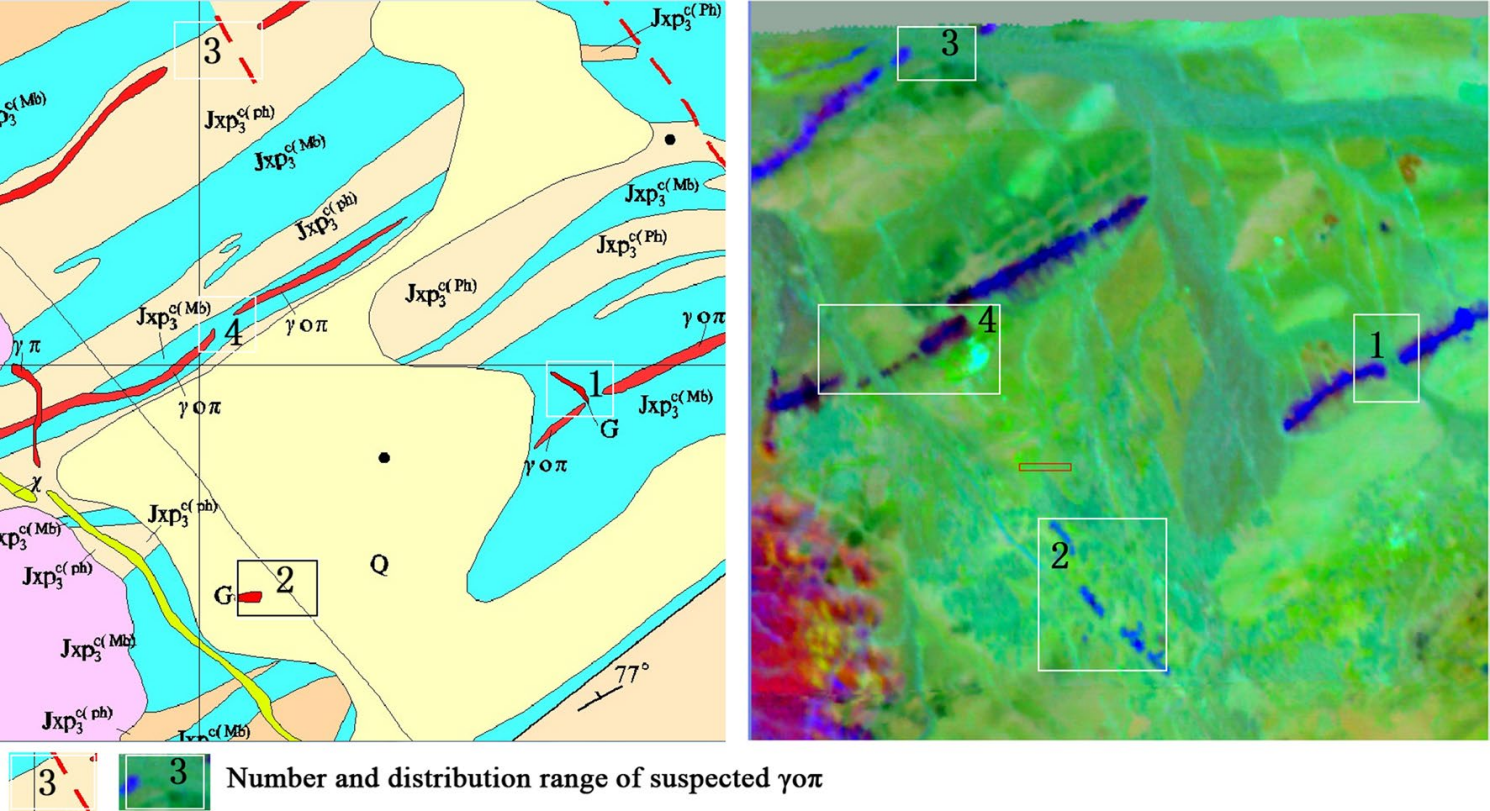
1:10,000 mineral geological map on the left and pseudo color composition of the 4th, 3rd and 6th components after MNF on the right. This figure is generated by Yu-Qing Wan, using ENVI (version4.3) created by the ENVI Team under an open license (http://www.enviidl.com/).

Figure 2 (right) shows the composition of the 4th, 3rd, and 6th MNF components, with γοπ highlighted. Positions 1, 2, 3, and 4 in the left figure are the places where γοπ is exposed. However, in the right figure, there is no complete patch in the corresponding position. Similarly, in the right figure, position 1 is the missing part of γοπ, and position 2 consists of four linear dark blue patches with suspected γοπ. The location and continuity of position 3 are significantly different from the left figure. In contrast, position 4 shows a significant displacement. All the blue stripes in the right figure are worth exploring, as a small fault appears to be staggered.

#### Mixture tuned matched filtering

**Figure 3:**
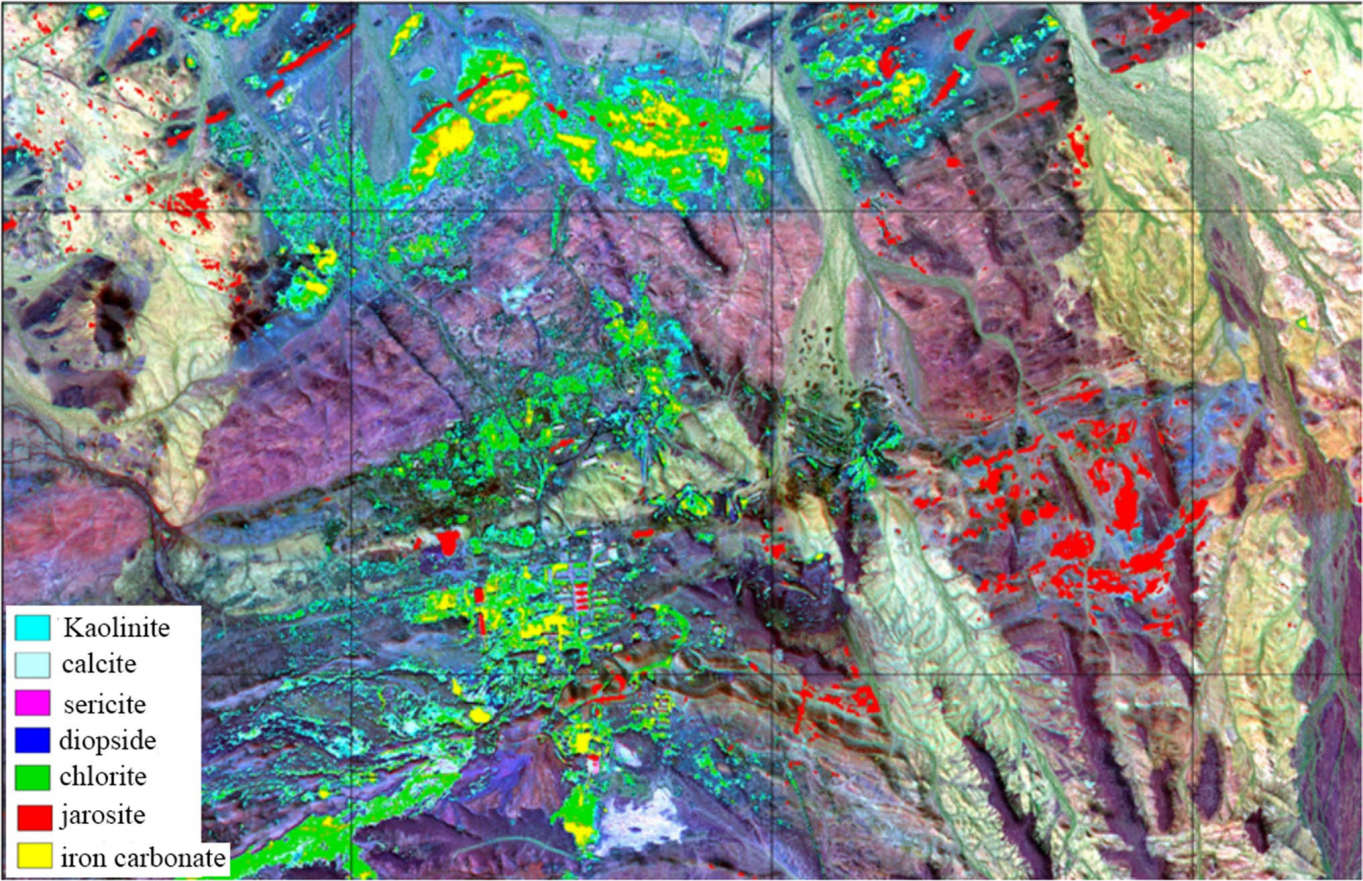
Piece of MTMF results. This figure is generated by Yu-Qing Wan, using ENVI (version4.3) created by the ENVI Team under an open license (http://www.enviidl.com/).

Mixture tuned matched filtering (MTMF) is a combination of mixture tuned and matched filtering techniques. It is the product of a combination of signal processing methodology and linear mixture tuned theory. The mixture tuned technique uses a linear spectrum mixing theory to limit the feasibility of mixing results and reduce the probability of false signals. MTMF uses the local separation technique to identify most end members. This technique uses a matched filtering technique to maximize response to known end member spectra, suppress the spectrum of unknown background compounds to match known spectrum signals, and provide a methodology to detect specific minerals by matching the spectrum library or image spectrum curve. Simultaneous to generating matched filter score images, MTMF generates an infeasible image based on the feasibility matrix between the composite background and the target spectrum. This reduces the number of false-positive pixels when using matched filters. Before performing the MTMF processing, we carefully read the distribution characteristics of the surface ore body and rock mass on the 1:10,000 geological mineral map, selected the key test area, and preset the mineral and rock spectra (seven types) that are likely to occur. We then performed the MTMF processing, and the results are shown in Fig. 3.

Figure 3 shows more Fe-rich carbonate rocks, jarosite, chlorite, kaolinite, and less sericite and calcite. The recognition results of these six types of rocks are reliable, but the diopside recognition results are too many to be reliable.

## Discussion and conclusion

### Discussion

1. Relationship between mineral group identification results of rock samples and measured spectra.

After spectral testing and field sample analysis, we selected highly suspicious ones and sent them to experts at Xi'an Center of Geological Survey to identify the mineral components using a polarized microscope and discover the reasons for changes in the spectral curve. Lamprophyre has the highest gold content of crustal igneous rocks^44^ and is also associated with a variety of minerals rich in molybdenum, gold, lead, zinc, copper, and antimony^45^. Many of the minerals mentioned above^39^ have also been found in this area. They are the focus and target of hyperspectral remote sensing research. As shown in Table 1, the classification accuracy of lamprophyre is very low and has no practical significance. Field investigation has shown significant differences in the composition, structure, surface color, and morphology of the lamprophyre veins in this area. Therefore, a series of lamprophyre samples were collected for spectral testing and microscopic identification. Table 2 shows some identification results of lamprophyre. Samples y057-1 and y057-2 in Table 2 are from adjacent sampling points, with slight surface weathering. Samples y058-1 and y058-2 are also from adjacent sampling points, indicating severe surface weathering.

**Table 2:** samples and analysis result of lamprophyre from Huaniushan.

Sample code	Name in field	Name under microscope	Composition (%)*
Y057-1	Lamprophyre, slight weathering	AMPL**	A:5–10% (C:7%, D:3%), B:85–90% (C:53%, D:37%)
Y057-2	Lamprophyre, slight weathering	AMPL**	A:5% (C:4%, D:1%), B:95% (D:55%, E:40%)
Y058-1	Lamprophyre, serious weathering	AMPL**	A:5–10% (C:8%, D:2%), B:85–90% (D:40%, E:30%, F:5%, G:5%, H:5–10%)
Y058-2	Lamprophyre, serious weathering	AMPL**	A:10–15%, (C:10%, D:3, I:2%), B:85–90% (D:40%, K:40%, L:5–10%)
Y062-2	Lamprophyre	GWASL***	J:70%, K:25–30%, L: < 1%
Y066-1	Lamprophyre	AMPL**	A:5% (C:4%, D:1%), B:95% (D:40%, E:40%, G:5–10%, L:5%)
Y080-1	Lamprophyre	AMPL**	A:10–15%, (C:10%, D:3%, I:2%), B:80–85% (D:65%, G:10%, F:5%, L:5–10%)

*A: phenocryst, B:matrix, C:augite, D: plagioclase, E: biotite, F: epidote, G: chlorite, H: calcite, I: quartz, J: Siliceous, K: sericite, L: ferruginous.

**AMPL:Alteration mica-plagioclase lamprophyre.

***GWASL:Gray white argillaceous siliceous slate.

**Figure 4:**
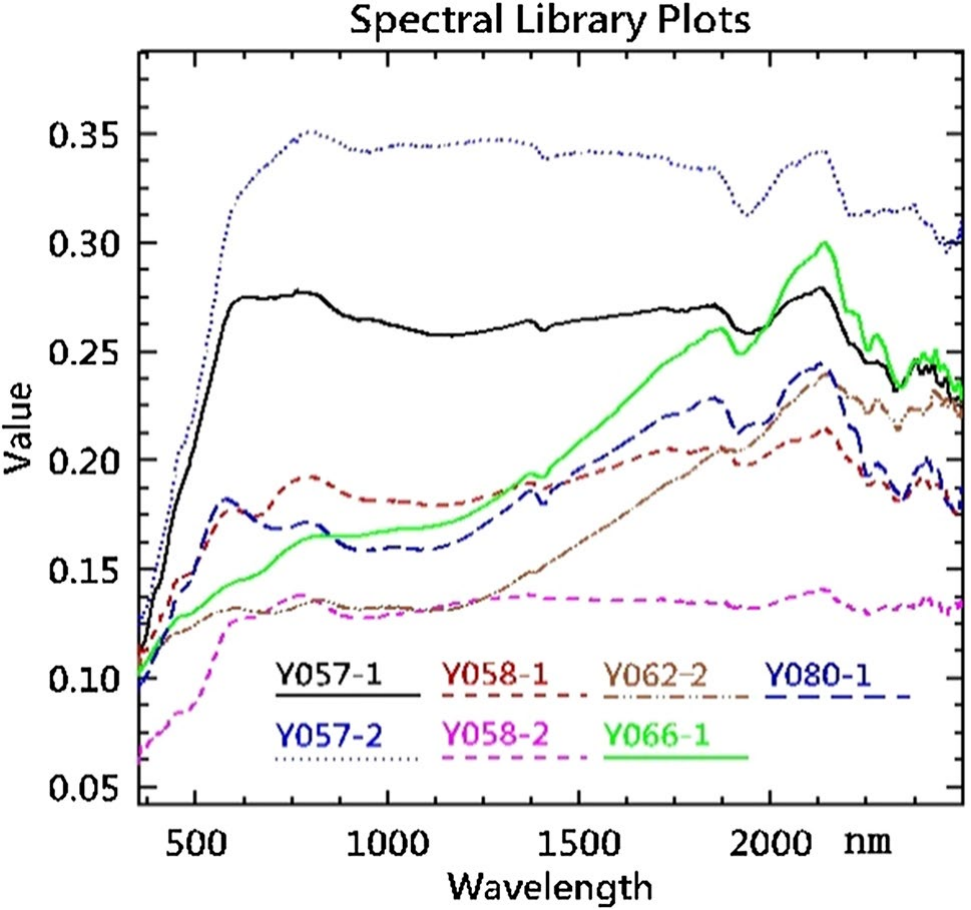
Shows the spectrum of 7 lamprophyre samples tested by ASD. This figure is generated by Yu-Qing Wan, using ENVI (version4.3) created by the ENVI Team under an open license (http://www.enviidl.com/).

Figure 4 shows the spectral curves of seven lamprophyre samples tested by ASD spectral meter. The sample code in Fig. 4 is the same as the sample code in Table 2. After identifying the y062-2 sample, the lamprophyre field identification results were rejected, but the spectral shape was similar to y066-1. It was found that the spectral curves and mineral composition of lamprophyres at different locations differed considerably from each other. Therefore, it is impossible to use only one spectral curve as the basis for the classification of lamprophyres in this region by hyperspectral images. Even in similar areas, it is necessary to subdivide it into multiple sub-areas according to changes in the position of adjacent strata and rock masses. It is also necessary to establish and classify sample training areas or deep learning areas, and finally merge the results of sub-areas.

(2) Selecting spectra.

Proir to Sam processing, it is necessary to select spectra of rocks or minerals known in the study area as the basis for discrimination. There are two more practical methods to obtain spectra. The first is to use a spectrometer to measure the spectra of known rocks and minerals. The second is to draw out the part of known rocks or minerals as the region of intrusting (ROIs) from the hyperspectral image, and then obtain the spectra from the ROIs.

Although the spectral purity from a spectrometer is high, the classification accuracy is often low due to the low spectral matching rate with the hyperspectral image. Moreover, a spectrometer’s spectrum must be resampled to match the wavelength and spectral interval of the hyperspectral image. In addition, the spectral calibration of hyperspectral images is difficult to meet theoretical requirements. Therefore, authors prefer to use the ROI spectrum for subsequent classification, provided that they have an adequate understanding of the actual situation in the field. To get a suit of efficient spectra, parts of ROIs need to be modified and supplemented to improve the OA (Kappa coefficient) based on the SAM recognition rate of main deposits (ore bodies).

**Figure 5:**
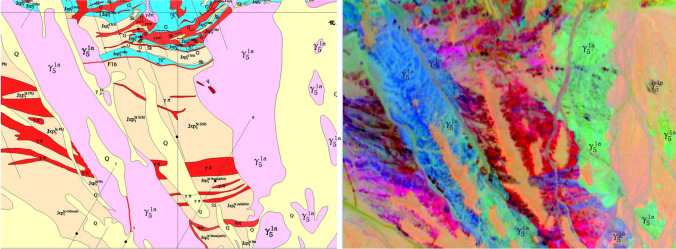
Mineral geological map on the left, composite image of the 2nd, 3rd and 14th components of MNF on the right. This figure is generated by Yu-Qing Wan, using ENVI (version4.3) created by the ENVI Team under an open license (http://www.enviidl.com/).

Figure 5 shows that as the mineral composition of the same set of rock or stratum changes, or the spatial position span increases, or it is alternately distributed with other rock masses, a single ROI and spectrum cannot be used as the only one standard, and it must be subdivided into multiple subunits for processing. After Sam processing, the subunits are combined into one set. As shown in Fig. 5 (left part), in the mineral geological map, γ51a granite body is widely distributed in this area, and contacts with various strata and rock masses, resulting in mineralization and alteration. The spectruma of γ51a in different parts change greatly. After MNF transformation and combination, the difference is very significant from Fig. 5 (right part), the color is from dark bluish-gray to light bluish-gray, bluish-green and dark green, bright green, even purplish red. These changes cannot be represented by only one spectraum. Otherwise the accuracy of classification and recognition is significantly reduced.

(3) Validation process.

**Figure 6:**
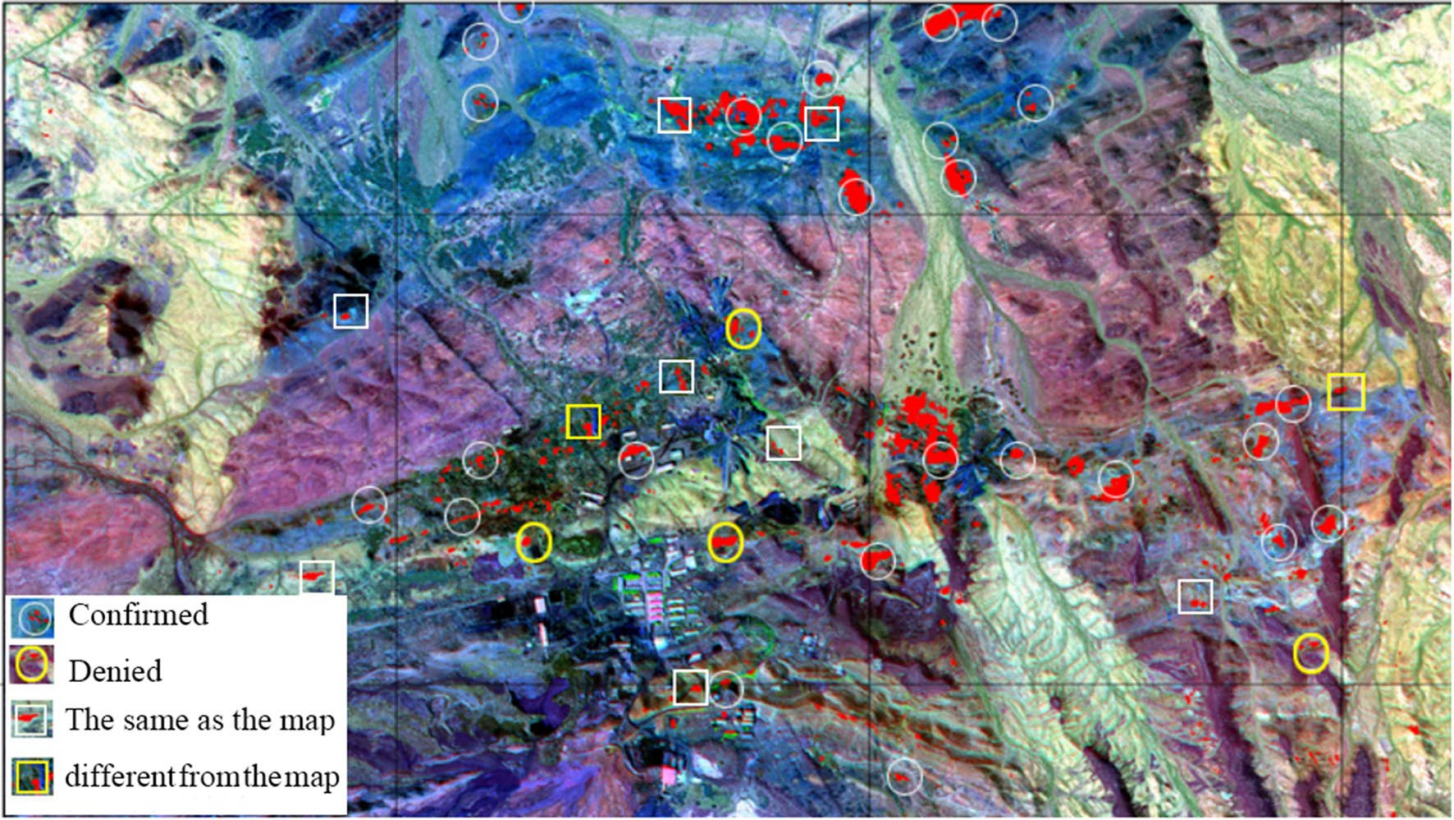
Distribution and results of verification points of deposit and mineralization information. This figure is generated by Mou-shun Jin, Yu-Hai Fan using ENVI (version4.3) created by the ENVI Team under an open license (http://www.enviidl.com/).

Two methods were used to validate the deposit information extracted from hyperspectral images. The first method was field validation. Ten potential mineralization targets were selected for field validation, eight of which had altered minerals, and two had no alteration (see Fig. 6). The second method was to refer to the 1:10,000 geological mineral map for validation. After identifying 30 iron-lead–zinc ore bodies on the map through enhancing and extracting hyperspectral image information, 26 of them had the same abnormal information as the map, but the other four did not. The results showed that hyperspectral images were valuable for extracting information about mineralization and alteration.

**Figure 7:**
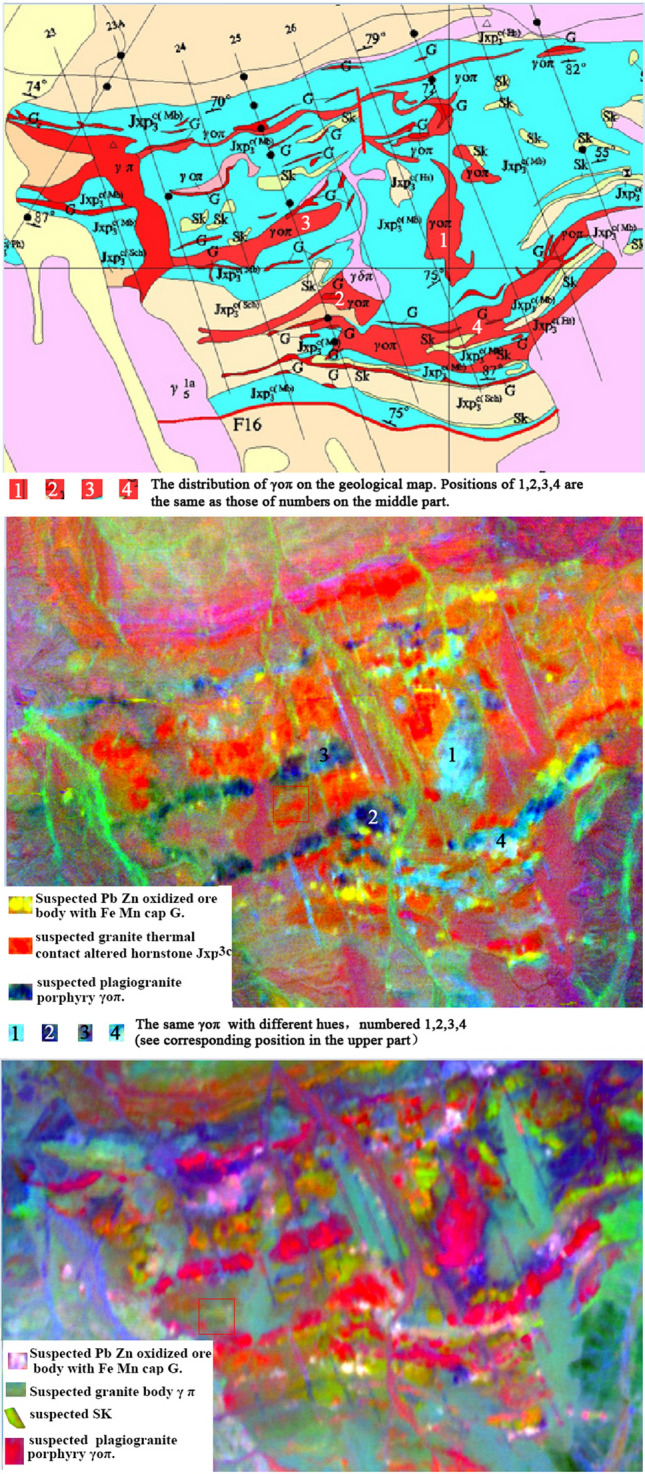
The upper is the mineral geological map of key areas, the middle is the 2nd, 3rd and 14th component combination of MNF, the lower is the 6th, 7th and 8th component combination of MNF. This figure is generated by Yu-Hai Fan, Mou-shun Jin using ENVI (version4.3) created by the ENVI Team under an open license (http://www.enviidl.com/).

The effective application of hyperspectral remote sensing technology in geological exploration is a long-term expectation of many geologists. This study effectively validated mineralization and alteration information in the rock mass and ore bodies with a 1:10,000 geological mineral map (see Fig. 7).

Figure 7 (upper part) is part of a 1:10,000 geological mineral map. The middle part shows a combination of the 2nd, 3rd, and 14th components after MNF. The main ore body, consisting of Pb–Zn oxidized ore bodies with a Fe–Mn cap (G), is clearly shown in the figure, corresponding to light yellow areas. This also reflects the color change of γοπ in different locations. For example, positions 2 and 3 in the figure are significantly different from positions 1 and 4, so it is worth further investigation. Figure 7 (lower part) shows a combination of the 6th, 7th, and 8th components of MNF, focusing on the effective reflection of Pb–Zn oxidized ore body with a Fe–Mn cap (G) and skarns (Sk). At the same time, most γοπ turns into red, but the large γ π rock mass does not. These two kind rocks have the same color in the upper left of Fig. 7 (upper part), it shows that this combination can effectively distinguish between γ π and γ o π, which is difficult by other methods effectively.

From an ore exploration perspective, the analysis of the geological mineral map shows that all G geological bodies in the region appear at the junction of γοπ and Jxp3c (MB) edges, with yellow spots in the middle and light pink spots in the lower parts of Fig. 7. Areas for subsequent exploration and validation are similar spots around known mining areas.

(5) Query for the accuracy of 1:10,000 geological mineral map.

After orthophoto correction and mosaicking, the aerial hyperspectral remote sensing image can meet the accuracy of 1:10,000 mapping. The spatial location of most rocks and features displayed on hyperspectral images can effectively match the geological mineral map. However, some strata boundaries and rock types do not match the geological mineral map. Figures 2 and 7 show that the same rock masses show significantly different hues and colors, while different types of rocks show similar hues. These chaotic phenomena require further field investigation and validation. Geological exploration itself is a process of continuous understanding, summarization, and a trusted approach. Existing geological maps inevitably have some errors and inadequate research. We should pay attention to suspicious information revealed by hyperspectral images and active investigation as they may be new mineral locations or deposits. There are many precedents for using hyperspectral remote sensing to edit geological maps, which is also an important future application direction.

## Conclusion

In the Huaniushan metal mining area, hyperspectral images are processed by spectral calibration and classification, and the results are compared to existing 1:10,000 geological mineral maps. Several effective methods extract and classify information, including SAM, MNF, PCA, and MTMF. Among them, the overall classification accuracy of SAM for 14 types of rocks is 65.37%, the Kappa coefficient is 0.59, and the recognition rate of Pb–Zn oxidized ore body with a Fe–Mn cap (G) is as high as 81%. The accuracy of the field investigation of the mineralized alteration zone and the ore body is more than 80%. Multiple components of the MNF, displayed in different combinations, significantly enhance the main formations and rock masses and improve recognition accuracy.

However, lamprophyres and skarns, which are closely related to many metallic minerals in this region, have poor recognition accuracy due to significant changes in mineral composition and spectral characteristics. It is necessary to subdivide lamprophyres and skarns into multiple subgroups, classify them based on spectral characteristics, and then merge them. It is also difficult to identify small dikes. Analysis of hyperspectral images and their classification results raises questions about 1:10,000 geological mineral maps because the colors of the same type of rock are different or the colors of different rock types are similar. In a nutshell, hyperspectral remote sensing is effective in identifying rocks (ore body). In addition, suspicious areas must be the focus of follow-up mineral resource research.
